# Low‐Field Magnetic Resonance Imaging of the Late Gestation Cervix and Birth Outcome Correlation: A Prospective Cohort Study

**DOI:** 10.1111/1471-0528.70103

**Published:** 2025-12-03

**Authors:** Simi Bansal, Alena U. Uus, Agnieszka Glazewska‐Hallin, Caitlin Allwin, Hadi Waheed, Vanessa Kyriakopoulou, Anna L. David, Dimitrios Siassakos, Manju Chandiramani, Jana Hutter, Lisa Story, Mary A. Rutherford

**Affiliations:** ^1^ School of Imaging Sciences and Biomedical Engineering, King's College London London UK; ^2^ Reseach Department for Early Life Imaging King's College London London UK; ^3^ Department of Women and Children's Health King's College London London UK; ^4^ Elizabeth Garrett Anderson Institute for Women's Health University College London London UK; ^5^ Smart Imaging Lab, Radiological Institute University Hospital Erlangen Erlangen Germany; ^6^ Fetal Medicine Unit, Guy's and St Thomas’ NHS Foundation Trust London UK

**Keywords:** birth outcomes, cervix, magnetic resonance imaging, pregnancy MRI

## Abstract

**Objective:**

To use low‐field MRI to produce reconstructions and 3D models of the cervix and to automate measurements for correlation with demographics and birth outcomes.

**Design:**

Prospective cohort study.

**Setting:**

KCL Advanced Imaging Centre, St Thomas's Hospital.

**Population:**

Late gestation (36‐41w) women attempting their first vaginal birth, recruited to the MiBirth study (*n* = 97).

**Methods:**

Reconstructed images were produced from 2D T2‐weighted Turbo‐Spin‐Echo 2D sequences acquired with a 0.55 T Freemax MRI scanner. Segmentations and anatomical landmarks were automated using an in‐house 3D deep learning segmentation network, from which cervical 2D measurements and 3D volumes were generated.

**Main Outcome Measures:**

Quality of reconstructed images and segmentations. Inter‐rater variability for cervical biometry. Correlation between cervical measurements, maternal demographics and birth outcomes.

**Results:**

Successful reconstructions were obtained for 92.9%; 84.9% were good quality. Excellent or good quality segmentations were obtained for all successful reconstructions (*n* = 99). Inter‐rater variability between automated and manual biometry was excellent or good for cervical measurements. Total cervical and stroma volumes significantly increased with cervical length (*p* < 0.01). Os diameters and utero‐cervical angle significantly decreased as cervical length increased (*p* < 0.001). Cervical stroma volume increased with maternal age (*p* = 0.02). Controlling for maternal age, an increased cervical volume was associated with an increased risk of caesarean section (OR 1.09, *p* = 0.04).

**Conclusions:**

This is a novel, accurate automated system to assess MRI late gestation cervical biometry and volumetry. We have shown that the late gestation cervical phenotype may influence birth outcomes and provided a new mechanism for increased risk of caesarean with maternal age.

## 1 Introduction

The cervix provides sufficient structural integrity to prevent spontaneous preterm birth yet can transform micro and macroscopically to allow fetal passage during labour. There is limited published information about the imaging phenotype or measurable changes of the late gestation cervix.

Cervical remodelling occurs at a microcellular level as it softens, shortens and dilates [1, 2, 3]. The extracellular matrix demonstrates collagen disorganisation [4, 5]. Cervical softening starts early in pregnancy, but ripening (thinning and shortening) before dilation, occurs hours to days before spontaneous labour [6]. Animal studies using diffusion MRI have shown early pregnancy changes in tissue organisation between the pregnant (E15–E18) and non‐pregnant murine cervix [7].

Characterisation of cervical changes is possible with physical examination, ultrasound and MRI. Digital qualitative assessment through vaginal examination with the Bishop's score is vulnerable to subjectivity and therefore unreliable [8, 9]. Cervical length measurement with transvaginal ultrasound scan (TVUS) is less subjective. Although 3D data can be obtained, the only parameter commonly assessed in clinical practice is cervical length [10, 11, 12, 13]. TVUS cervical length measurement at term has some value in predicting onset of labour and birth mode [14, 15, 16, 17, 18, 19]. Mid‐gestation short cervical length and increased utero‐cervical angle are useful in predicting preterm birth [20, 21, 22, 23].

MRI provides superior soft tissue contrast to ultrasound and provides functional as well as structural information [24]. It allows for automated measurements and segmentations, overcoming the manual processing challenges of inter‐observer variability and time‐consuming segmentation. Low‐field scanners have a wide bore, so they are suitable to accommodate women in late gestation and offer improved participant comfort when compared with 1.5 and 3 T scanners [25]. Low‐field MRI also has the benefit of reducing both artefacts from B0 inhomogeneities and distortions from B1 inhomogeneities, as well as providing a large homogeneous field enabling whole uterus scanning [25].

MRI has shown a minor increase in cross‐sectional area and cervical stroma signal intensity with increasing gestation [26]. Diffusion weighted and tensor imaging have shown changes in apparent diffusion coefficient (ADC), suggesting collagen fibre disorganisation with advancing gestation [27]. Higher signal intensity in the cervical stroma may be associated with birth outcome [28, 29]. Changes in MR diffusion imaging are seen in women who experienced preterm birth and are correlated with delivery timing [30].

Using data from the MiBirth study, which aims to predict birth outcomes using MRI in late gestation, we investigate the feasibility of low field (0.55 T) MRI of the cervix. The study objective is to show that 0.55 T MRI of the late gestation cervix produces good quality reconstructions and 3D models. We describe a novel protocol for manual and automated cervical measurements to define the late gestation phenotype. We relate automated 2D biometry and 3D volumetry with birth outcomes for a late gestation cohort (*n* = 90) for the first time. Cervical measurements are compared with maternal demographics that may affect cervical structure, such as maternal age, ethnicity and spontaneous preterm birth risk factors (smoking, previous cervical surgery) [31, 32, 33]. Cervical measurements are compared with birth outcomes to see if changes observed in preterm birth are also present at term birth [20, 23]. We hypothesise that emergency caesarean section or induction of labour may be required in part due to failure of the cervix to remodel appropriately.

## 2 Methods

### 2.1 Datasets and Preprocessing

As part of the prospective MiBirth study (REC 23/LO/0685), low risk women planning their first vaginal birth, or VBAC consented to have a 0.55T MRI scan (MRI protocol in Appendix S1). T2‐weighted Turbo‐Spin‐Echo sequences of the cervix were obtained. The MiBirth study has an active Patient and Public Involvement group that advise on improvements and future directions for the study. The initial cohort contained 99 pregnant women between 35+5 and 40+1 weeks gestational age (GA). Of these, 8 women had repeat scans 1‐3 weeks after their initial MRI, creating 105 datasets. Failed reconstructions (*n* = 6) were escluded, resulting in 99 datasets (Figure S1). Birth outcome data, including onset mode of birth was available for all participants; women who opted for an elective caesarean section after participation in the study were excluded from birth outcome analysis (*n* = 9). Interval to onset of labour data was available for 68 participants. All women underwent scanning on a 0.55T Free.Max MR scanner at the KCL Advanced Imaging Centre, St Thomas's Hospital. Antenatal and birth data were collected from clinical notes and stored on a Redcap database [34, 35].

Images from a separate smaller cohort of 20 women, who had a 0.55 T MRI scan between 33+4 and 40+2 weeks gestation as part of the MEERKAT study (REC 21/LO/0742), were used for intra and inter‐rater variability for manual cervical biometry.

### 2.2 3D Image Reconstruction

The 3D images of the cervix were reconstructed using the DSVR method [36] in SVRTK toolbox to 0.8 mm resolution with uterus sagittal stack as a template and automated deep learning masking based on a pre‐trained in‐house 3D UNet [37]. Images (*n* = 99) were scored for quality by a clinician (SB) with 1 years' experience in MR cervix image analysis on numerical scale (1= failed, 2 = poor, 3 = acceptable, 4 = good). Datasets with poor or failed reconstructions due to unexpected maternal positional change (due to maternal discomfort) during the scan (*n* = 6), were excluded from quality and statistical analysis.

### 2.3 3D Cervix Volumetry

#### 2.3.1 Segmentation Protocol

**FIGURE 1:**
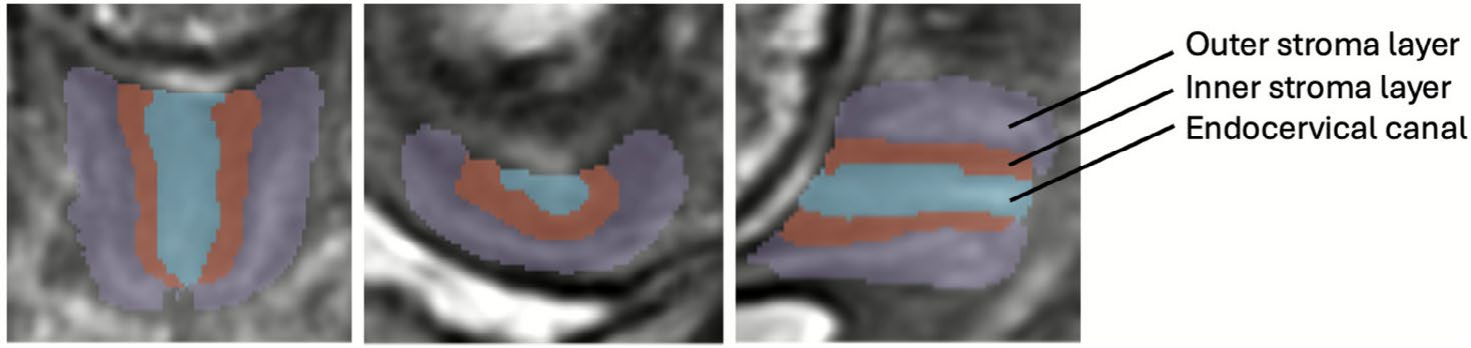
Axial, coronal and sagittal reconstructions and segmentations; purple = outer stomal layer, red = inner stroma layer, blue = cervical canal.

**FIGURE 2:**
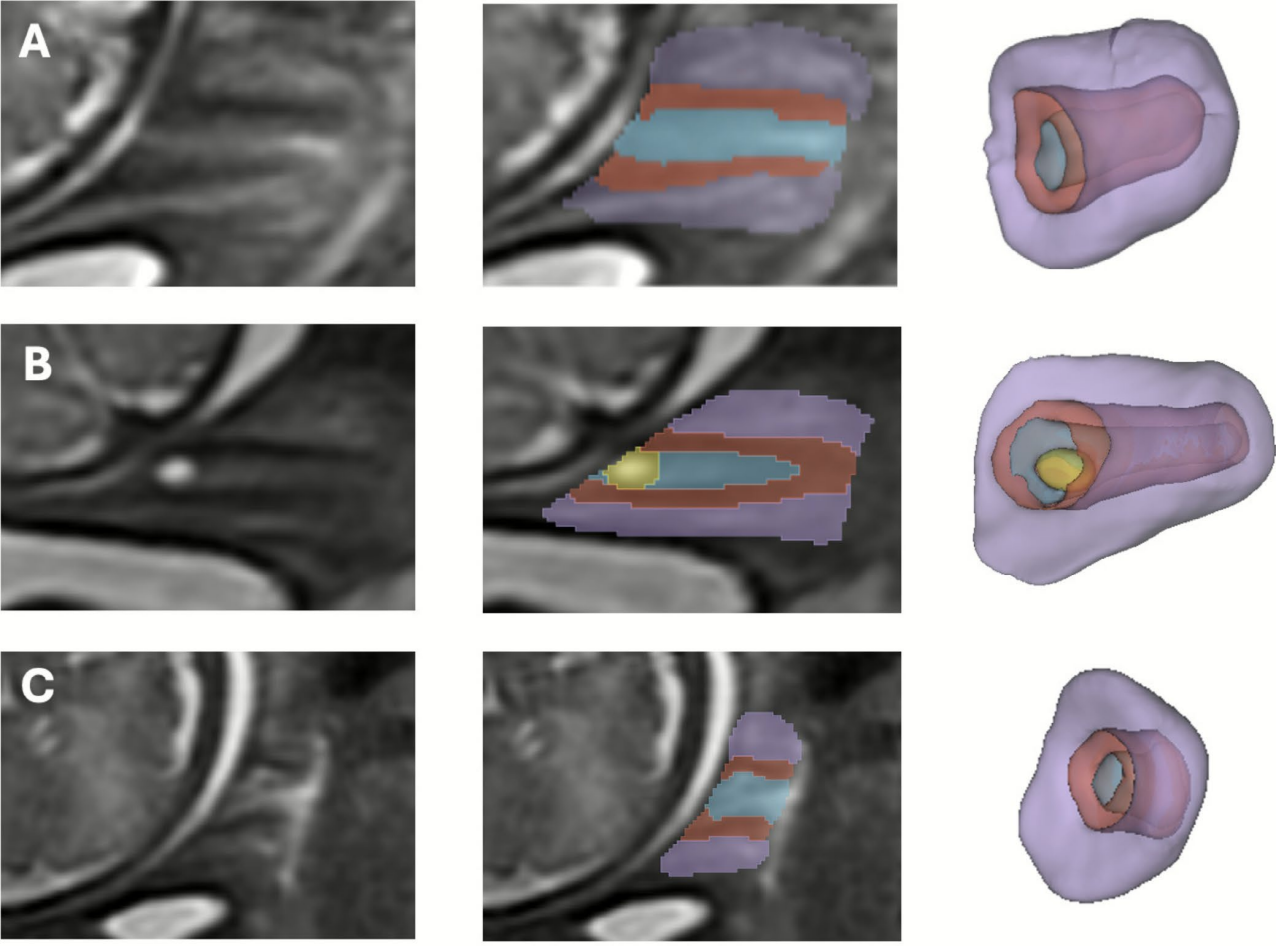
Process of reconstruction, segmentation and model creation: (A) Typical cervix, (B) Cervix with an endocanal cyst, (C) Short cervix.

The protocol for segmentation was defined by clinicians (LS, AGH, SB) with 1–4 years' experience in fetal and cervical MRI using ITK‐SNAP [38] in 3D DSVR images. The segmented regions include three cervical layers (outer stroma, inner stroma and canal) and canal cysts if present, as illustrated in Figures 1 and 2 and Video S1.

#### 2.3.2 Automated Segmentation

The network was trained to automatically segment structures (Appendix S1). Segmentations (*n* = 99) were scored for quality by (AU) with 4 years' experience in MR cervix image analysis with a numerical scale (1 = poor, 2 = moderate, 3 = good, 4 = excellent, definitions in Table S1). Poor and failed segmentations were excluded from quality and statistical analysis (*n* = 6). In all of these cases there was an unexpected maternal positional change during the scan or a short cervix, both affecting reconstruction quality.

### 2.4 2D Cervical Biometry

#### 2.4.1 Manual Measurement Protocol

A protocol for standardising measurements of the cervix was defined, based on existing literature (Table S2) [19, 20, 29, 39, 40, 41]. Measurements were taken in 3D slicer. Intra‐rater variability for manual cervical biometry measurements, including between 2‐ and 3‐point cervical length, was calculated on the smaller cohort of 20 women (gestational ages 33+4 to 40+2 weeks) by 2 experienced raters (CA, SB).

#### 2.4.2 Automated Biometry

The biometric measurements defined in Table S2 were automated by using in‐house 3D deep learning segmentation network of the cervix and morphological operations combined in one script. The automated pipeline was evaluated vs. manual biometry measurements for 20 cases performed by 3 experienced raters (AGH, AU, SB) and inter‐rater variability was calculated. Average manual measurements were compared with automated measurements for 20 cases.

### 2.5 Statistical Analysis

Data organisation was performed in Microsoft Excel. Datasets were tested for normality prior to analysis using Shapiro‐Wilks in R. Inter‐rater correlations were calculated using SPSS and R. Generalised linear regression models (GLMs) were created in R. Outliers (values 1.5 times the interquartile range of Q1 and Q3) were removed from generalised linear models.

## 3 Results

### 3.1 Demographics

Maternal age ranged from 19 to 42 and BMI ranged from 18 to 32. A large range of ethnicities were included, with the majority being White. Most women were nulliparous and very few had risk factors for spontaneous preterm birth (Table S3).

### 3.2 Quality of Reconstructions and Segmentations

Successful reconstructions were obtained for 92.9% of cases (*n* = 105). Of those 84.9% were good/acceptable quality. Automated segmentations were obtained for cases where reconstruction did not fail and 100% of these were excellent or good quality (Figure S2).

### 3.3 Cervical Biometry

#### 3.3.1 Reliability of Measurements

Inter‐rater variability for manual cervical biometry measurements between 3 raters was excellent or good (Table S4). Variability between manual 2‐ and 3‐point cervical length measurements was excellent (correlation coefficient 0.89, CI 0.75–0.96). Intra‐rater reliability between average manual and automated cervical biometry measurements was excellent (Table S5).

#### 3.3.2 Cervical Biometry Data and Correlation With Demographics

Automated MRI measurements of cervical biometry are shown in Table S6. For most women (81.8%) the diameter of the internal os was larger than the external os (Figure S3). Mean difference in os diameters was 3.5 mm (range −4.6 to 17.7 mm).

Total cervical and stroma volumes significantly increased with cervical length (*p* < 0.01, Figure S4). Internal and external os diameters and utero‐cervical angle significantly decreased as cervical length increased (*p* < 0.001, Figure S4). Canal volume tended to increase with cervical length (*p* = 0.07, Figure S4). There was no significant correlation between cervical measurements and gestational age (35+5 to 40+1) (Figure S5).

**FIGURE 3:**
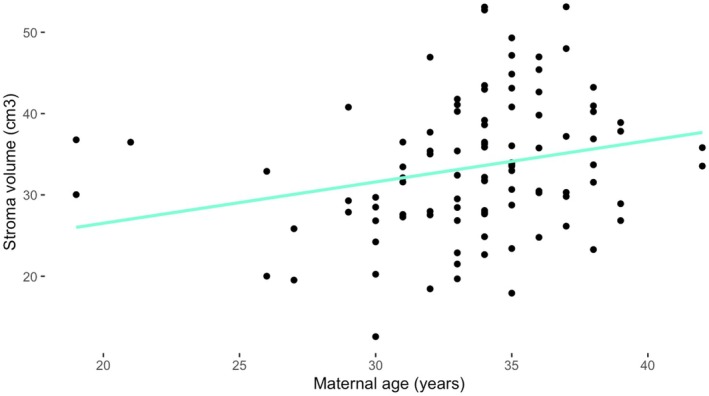
Cervix stroma volume compared with maternal age.

Cervical stroma volume increased with maternal age (*p* = 0.02; Figure 3) There was no significant correlation between the other cervical measurements and maternal demographics. There was no correlation between cervical length and spontaneous preterm birth risk factors (smoking history, previous cervical surgery).

For the 7 cases with repeat scans, cervical length, total cervical volume and stroma volume decreased or remained static (Table S7). Os diameters increased or remained static (Figure S6).

### 3.4 Birth Outcomes

Of the initial 99 women, 8 opted for elective caesarean section, so they were excluded from birth outcome analysis. Birth outcome data are shown in Table S8.

Black ethnicity potentially increased risk of caesarean section but the evidence was weak (Figure S7, *n* = 6, odds ratio 18.2, CI 1.18–666.6, *p* = 0.06). Demographics were not related to chance of requiring induction of labour (Figure S8).

#### 3.4.1 Correlation of Birth Outcome With Cervical Measurements

**FIGURE 4:**
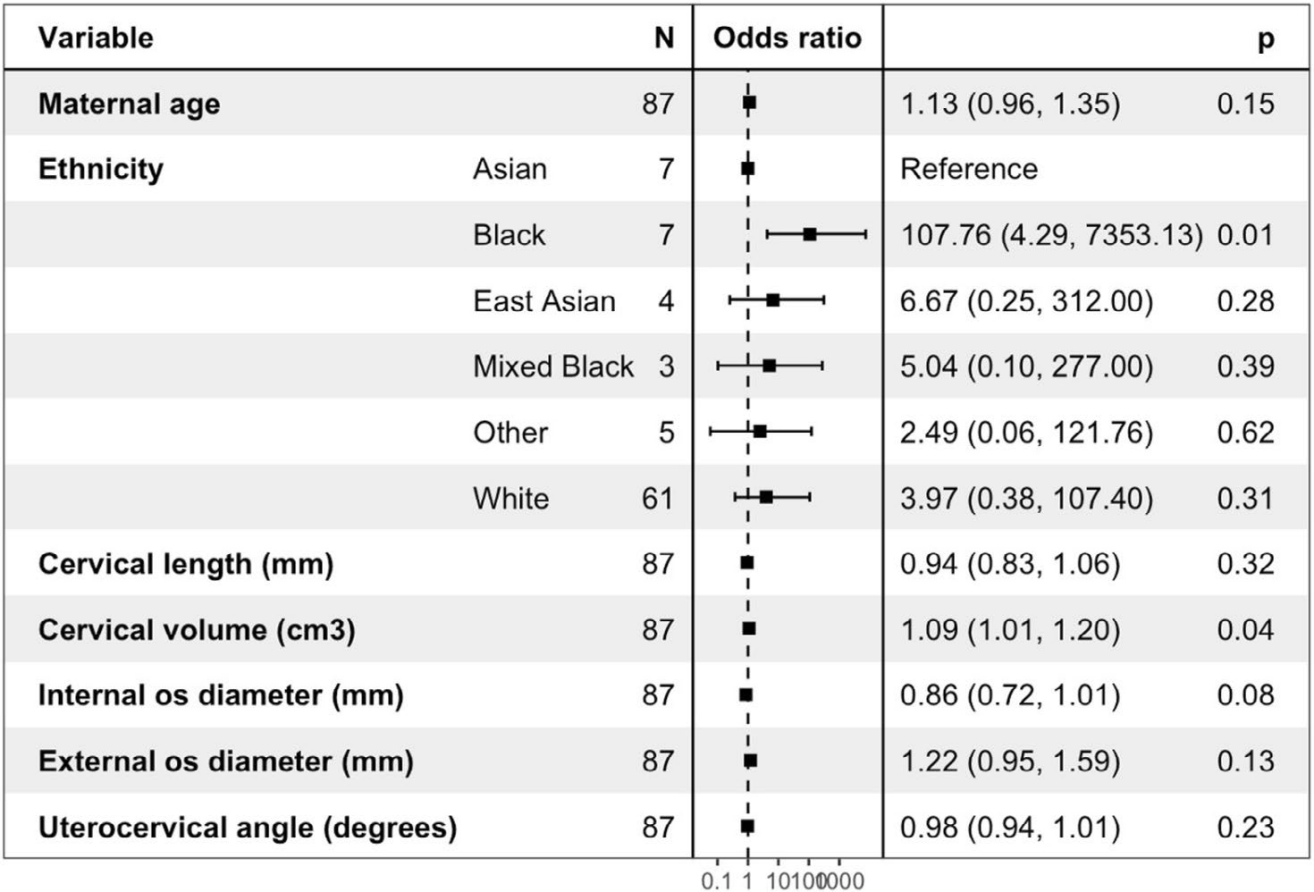
Forest plot with odds ratios for chance of caesarean section with cervical measurements.

When controlling for maternal age and ethnicity using GLMs, an increased cervical volume at the time of MRI was associated with an increased risk of caesarean section (odds ratio 1.09, *p* = 0.04) (Figure 4).

GLMs for cervical measurements showed that the risk of requiring induction of labour (for any indication, corrected for gestational age at MRI) was potentially associated with a smaller external os diameter, but the evidence was weak (odds ratio 0.83, CI 0.66–1.03, *p* = 0.09) (S5). There was no correlation between cervical measurements with length of labour (from start of induction to end of first stage), or successful vaginal birth after induction of labour.

## 4 Discussion

### 4.1 Main Findings

We have shown that MRI cervix at 0.55 T field strength is acceptable and feasible in late gestation pregnancy and produces high quality 3D reconstructions of the cervix. Where reconstructions failed, this was generally due to unexpected positional deviations during the scan. High quality automated segmentations were produced by the trained network for all adequate/good quality reconstructions, allowing for landmark localisation and automated 2D measurements.

We have defined a reproducible protocol for manual cervical biometry, as evidenced by the high intra‐rater agreeability. Automated and manual 2D biometry measurements, including between 2‐ and 3‐point cervical length, were well correlated. Therefore, these automated measurements are deemed reliable to use for ongoing analysis.

We have shown that in late pregnancy lower cervical volume is associated with shorter cervical length and have demonstrated that is due to an increase in stroma rather than canal volume (Figure S4). The change in stroma volume is likely to represent microcellular reorganisation of the extracellular matrix. Our preliminary data also show that a larger stroma volume increases chance of caesarean section (Figure 4). We have shown that both internal and external os diameters decreased with cervical length, supporting the premise that the cervix dilates as it shortens. For most participants, the internal os diameter was larger than the external os, suggesting that internal os dilation may occur first (Figure S3). This mirrors cervical funnelling seen in women at increased risk of spontaneous preterm birth, where the internal os is dilated and the external os remains closed.

Cervical biometry and volumetry in general were not related to maternal demographics, smoking history or previous cervical surgery, potentially due to lack of heterogeneity in our study cohort.

Biometry and volumetry did not significantly change within the limited gestational age range in this study. This is in keeping with the literature [42, 43]. This suggests that cervical macrostructural change occur rapidly, during or just prior to labour. However, in a small number of cases where repeat data was obtained, changes in the cervical length and internal os were seen, suggesting that for some women macrostructural changes may start before labour (Figure S6). Additionally, microstructural changes may precede measurable microstructural changes. Further serial data analysis is needed on a larger cohort to confirm these findings.

### 4.2 Interpretation

We did not find any correlation between cervical length or internal os diameter and risk of requiring induction of labour. This is in keeping with some ultrasound studies [10] and may be as we are measuring too soon to see any changes [13, 18]. Moreover in this small cohort the number of inductions for postdates was too small to comment on.

Our study potentially supports data in the literature showing chance of delivery by emergency caesarean section increases with maternal age, but evidence was weak [44, 45]. The reason for this increased risk is likely multifactorial. Advanced maternal age is an independent risk factor for various pregnancy complications, including gestational diabetes and hypertension, which are associated with increased chance of caesarean [46, 47]. Physiological explanations for this risk generally focus on ineffective contractions, due to an ageing myometrium or reduced oxytocin receptors [44]. Our preliminary data provides a new factor to consider, as we have shown that cervical stroma volume significantly increases with maternal age (Figure 3). In the mouse, the cervical stroma is known to become more fibrous or hyaline with maternal age, which may explain this increase [31, 32]. We propose that this cervical structure change with age may reduce its ability to remodel as required for labour to start or progress.

### 4.3 Strengths and Limitations

The study cohort are ethnically diverse, reflecting our obstetric population. Delivery outcomes are similar to nationally acquired data. We have shown that automation of cervical measurements on low field MRI is possible. High inter‐rater variability between automated and manual measurements suggests the automated measurements are reliable.

Whilst imaging at low field has the advantages of acceptability and comfort and increases in field homogeneity, signal to noise ratio is reduced. Reconstructions may therefore be less accurate than at higher field strengths, and landmarks harder to define and reproduce due to similarities in signal intensities between different tissue types. There is a low sample size for some variables, including extremes of age and some ethnicities.

## 5 Conclusions

We have developed an efficient and accurate system for performing cervical biometry and volumetry, that is both automated and using 0.55 T field‐strength MRI for the first time. We have described a reliable protocol for measuring cervix biometry and volumetry. We have demonstrated that cervical volume impacts upon risk of caesarean section, and that cervical changes can occur prior to labour onset and delivery. We have demonstrated the impact of age on the cervical stroma volume and provided a hypothesis for its effect on caesarean section risk. We have shown that late gestation cervical phenotype may play a role in prediction of birth outcome.

This study provides a basis for the analysis of vast amounts of MRI cervix data in relation to birth outcomes in large cohorts, for example the 500 women to be recruited as part of the MiBirth study. Associations between cervix biometry and volumetry with birth outcomes may form part of a larger algorithm to better predict mode of birth.

Future studies need to assess cervical measurements in a larger cohort to reach reliable conclusions about correlations with demographics and delivery outcomes. The addition of diffusion information from T2* images of the cervix may also provide more information about how the cervix remodels leading up to labour, and if changes impact birth outcomes [33].

## Author Contributions

**S.B.:** conceptualisation, data curation, formal analysis, methodology, writing – original draft. **A.U**.: conceptualisation, data curation, formal analysis, methodology, writing – original draft. **A.G.H.:** data curation, formal analysis. **C.A.:** data curation, formal analysis. **H.W.:** data curation. **V.K.:** data curation, project administration. **A.D.:** funding acquisition, writing – review and editing. **D.S.:** funding acquisition, writing – review and editing. **M.C.:** funding acquisition, supervision, writing – review and editing. **J.H.:** data curation, funding acquisition, writing – review and editing. **L.S.:** data curation, funding acquisition, writing – review and editing. **M.R.:** conceptualisation, funding acquisition, supervision, writing – review and editing.

## Funding

This work was supported by the Medical Research Council (MR/X010007/1), the NIH Human Placenta Project grant (1U01HD087202‐01), the NIHR Clinical Research Facility (CRF) at Guy's and St Thomas' and by the NIHR Biomedical Research Centre based at Guy's and St Thomas’ NHS Foundation Trust and King's College London. L.S. is funded by Health Education England/National Institute for Health Research (NIHR) (NIHR Advanced Fellowship 301664). J.H. is funded by DFG Heisenberg funding (502024488) and an ERC StG EARTHWORM (101165242). A.L.D. is part funded by the NIHR University College London Hospitals Biomedical Research Centre. The views expressed are those of the authors and not necessarily those of the NHS, the NIHR or the Department of Health.

## Ethics Statement

MiBirth Study—London‐Harrow Research Ethics Committee, REC 23/LO/0685, 18/9/2023. MEERKAT Study—London—Bromley Research Ethics Committee, REC 21/LO/0742, 8/12/2021.

## Conflicts of Interest

The authors declare no conflicts of interest.

## Supporting information


**Appendix S1:** Supporting information.
**Figure S1:** Flowchart of number of women and scans included in data analysis.
**Figure S2:** Quality control: (A) Visual scale for grading quality of reconstructions, (B) Qualitative evaluation of 0.55 T cervix reconstructions, rated good, acceptable, poor or failed. (C) Qualitative evaluation of 0.55 T cervix segmentations, rated excellent, good, acceptable or poor.
**Figure S3:** Bar chart showing the external os diameter subtracted from the internal os diameter (mm).
**Figure S4:** Cervical length compared with other cervical measurements: (A) Total cervical volume, (B) Stroma volume, (C) Canal volume, (D) Internal os diameter, (E) External os diameter, (F) Utero‐cervical angle, *p < 0.01.
**Figure S5:** Cervical measurements with gestational age: (A) Cervical length, (B) Internal os, (C) External os, (D) Utero‐cervical angle, (E) Stroma volume, (F) Canal volume.
**Figure S6:** Paired biometry measurements: (A) Cervical length, (B) Stroma volume, (C) Canal volume, (D) Internal os diameter, (E) External os diameter, (F) Utero‐cervical angle.
**Figure S7:** Forest plot with odds ratios for risk of caesarean section with demographics.
**Figure S8:** Forest plot with odds ratios for risk of induction of labour with cervical measurements.
**Table S1:** Definitions for segmentation quality control scoring.
**Table S2:** Formalised measurement definitions for the proposed landmark‐based cervix biometry protocol.
**Table S3:** Demographic data of participants.
**Table S4:** Intraclass correlation coefficients and interpretation for manual cervical measurements by 3 raters.
**Table S5:** Intraclass correlation coefficients and interpretation for average manual and automated cervical measurements.
**Table S6:** MRI automated cervical measurements.
**Table S7:** Change in cervical biometry in paired scans.
**Table S8:** Delivery outcomes.
**Video S1:** Axial, coronal and sagittal reconstructions with overlying segmentations shown in 3D Slicer; purple = outer stomal layer, red = inner stroma layer, blue = cervical canal.

## Data Availability

The individual fetal MRI datasets used for this study are not publicly available due to ethics regulations.
