# Supplementary material for: Low‐Field Magnetic Resonance Imaging of the Late Gestation Cervix and Birth Outcome Correlation: A Prospective Cohort Study
Source: BJOG. 2025 Dec 3;133(6):1166–73. doi: 10.1111/1471-0528.70103 (PMC13040424; doi:10.1111/1471-0528.70103)
Supplement: Supplementary file 1 — Appendix S1: Supporting information. Figure S1: Flowchart of number of women and scans included in data analysis. Figure S2: Quality control: (A) Visual scale for grading quality of reconstructions, (B) Qualitative evaluation of 0.55 T cervix reconstructions, rated good, acceptable, poor or failed. (C) Qualitative evaluation of 0.55 T cervix segmentations, rated excellent, good, acceptable or poor. Figure S3: Bar chart showing the external os diameter subtracted from the internal os diameter (mm). Figure S4: Cervical length compared with other cervical measurements: (A) Total cervical volume, (B) Stroma volume, (C) Canal volume, (D) Internal os diameter, (E) External os diameter, (F) Utero‐cervical angle, *p < 0.01. Figure S5: Cervical measurements with gestational age: (A) Cervical length, (B) Internal os, (C) External os, (D) Utero‐cervical angle, (E) Stroma volume, (F) Canal volume. Figure S6: Paired biometry measurements: (A) Cervical length, (B) Stroma volume, (C) Canal volume, (D) Internal os diameter, (E) External os diameter, (F) Utero‐cervical angle. Figure S7: Forest plot with odds ratios for risk of caesarean section with demographics. Figure S8: Forest plot with odds ratios for risk of induction of labour with cervical measurements. Table S1: Definitions for segmentation quality control scoring. Table S2: Formalised measurement definitions for the proposed landmark‐based cervix biometry protocol. Table S3: Demographic data of participants. Table S4: Intraclass correlation coefficients and interpretation for manual cervical measurements by 3 raters. Table S5: Intraclass correlation coefficients and interpretation for average manual and automated cervical measurements. Table S6: MRI automated cervical measurements. Table S7: Change in cervical biometry in paired scans. Table S8: Delivery outcomes. Video S1: Axial, coronal and sagittal reconstructions with overlying segmentations shown in 3D Slicer; purple = outer stomal layer, red = inner stroma layer, blu [file BJO-133-1166-s001.zip › bjo70103-sup-0001-AppendixS1.docx]

Appendix S1 – MRI image acquisition and reconstruction protocol

MiBirth MRI protocol

Women were recruited at St. Thomas and University College London hospitals. Recruitment criteria included women with singleton pregnancies planning their first vaginal birth (including VBAC) who had no contraindications to MRI. MRI was performed with an empty bladder in left lateral for 7-8 minutes, then in supported supine (approximately 45° anterior supine tilt) for approximately 1 hour. BP was monitored every 10 minutes. Heart rate and oxygen saturations were monitored continuously. All procedures were performed in accordance with relevant guidelines and regulations. Informed written consent was obtained from all participants. In total, 10 participants had a positional deviation due to discomfort, and 1 participant could not complete the scan due to claustrophobia; this participant was excluded from the main cohort.

Image acquisition

Study images were acquired using a 0.55T Siemens MAGNETOM Free.Max MRI system with 6-element flexible coil and a 9-element spine coil built into the patient table using a dedicated low field fetal acquisition protocol.^43^ The structural T2w HASTE stacks were acquired with TE=105ms, acquisition resolution 1.48 x 1.48mm, slice thickness 4.5mm, 0mm gap and 3-6 whole uterus stacks covering the uterus in axial/coronal and sagittal orientation

Automated segmentation

The network for 3D segmentation the cervix in 3D DSVR MRI reconstructions was created in MONAI^44^ framework based on the standard 3D UNet architecture^33^ with five encoder-decoder blocks (output channels 32, 64, 128, 256 and 512). We used the standard MONAI configuration for training, AdamW optimiser, combined Dice and cross entropy loss training and bias field and affine rotation augmentations. The training was performed in three stages based on manual refinement of pretrained network outputs. Initially, a set of labels for 20 cases with varying anatomy and acquisition protocols were manually created (by AGH, SB and AU) in ITK-SNAP based on the proposed segmentation protocol. The segmentation label files include 5 labels: canal inner os, outer os, cysts and the bladder. They were then used to pretrain the network followed by manual refinement for another set of 20 cases to increase the dataset and used further retraining. The final version of the network was trained on 60 datasets. Preprocessing included resampling with padding to 128x128x128 grid and intensity scaling to 0-1 range. After segmentation the volumes of the labels were automatically extracted for all cases.
