# Supplementary material for: Low‐Field Magnetic Resonance Imaging of the Late Gestation Cervix and Birth Outcome Correlation: A Prospective Cohort Study
Source: BJOG. 2025 Dec 3;133(6):1166–73. doi: 10.1111/1471-0528.70103 (PMC13040424; doi:10.1111/1471-0528.70103)
Supplement: Supplementary file 1 — Appendix S1: Supporting information. Figure S1: Flowchart of number of women and scans included in data analysis. Figure S2: Quality control: (A) Visual scale for grading quality of reconstructions, (B) Qualitative evaluation of 0.55 T cervix reconstructions, rated good, acceptable, poor or failed. (C) Qualitative evaluation of 0.55 T cervix segmentations, rated excellent, good, acceptable or poor. Figure S3: Bar chart showing the external os diameter subtracted from the internal os diameter (mm). Figure S4: Cervical length compared with other cervical measurements: (A) Total cervical volume, (B) Stroma volume, (C) Canal volume, (D) Internal os diameter, (E) External os diameter, (F) Utero‐cervical angle, *p < 0.01. Figure S5: Cervical measurements with gestational age: (A) Cervical length, (B) Internal os, (C) External os, (D) Utero‐cervical angle, (E) Stroma volume, (F) Canal volume. Figure S6: Paired biometry measurements: (A) Cervical length, (B) Stroma volume, (C) Canal volume, (D) Internal os diameter, (E) External os diameter, (F) Utero‐cervical angle. Figure S7: Forest plot with odds ratios for risk of caesarean section with demographics. Figure S8: Forest plot with odds ratios for risk of induction of labour with cervical measurements. Table S1: Definitions for segmentation quality control scoring. Table S2: Formalised measurement definitions for the proposed landmark‐based cervix biometry protocol. Table S3: Demographic data of participants. Table S4: Intraclass correlation coefficients and interpretation for manual cervical measurements by 3 raters. Table S5: Intraclass correlation coefficients and interpretation for average manual and automated cervical measurements. Table S6: MRI automated cervical measurements. Table S7: Change in cervical biometry in paired scans. Table S8: Delivery outcomes. Video S1: Axial, coronal and sagittal reconstructions with overlying segmentations shown in 3D Slicer; purple = outer stomal layer, red = inner stroma layer, blu [file BJO-133-1166-s001.zip › bjo70103-sup-0010-TableS1.docx]

| **Segmentation quality** | **Definition** |
| --- | --- |
| Excellent | No manual refinement required |
| Good | Minimal manual refinement required |
| Moderate | Moderate manual refinement required |
| Poor | Significant segmentation errors |
