# Supplementary material for: Low‐Field Magnetic Resonance Imaging of the Late Gestation Cervix and Birth Outcome Correlation: A Prospective Cohort Study
Source: BJOG. 2025 Dec 3;133(6):1166–73. doi: 10.1111/1471-0528.70103 (PMC13040424; doi:10.1111/1471-0528.70103)
Supplement: Supplementary file 1 — Appendix S1: Supporting information. Figure S1: Flowchart of number of women and scans included in data analysis. Figure S2: Quality control: (A) Visual scale for grading quality of reconstructions, (B) Qualitative evaluation of 0.55 T cervix reconstructions, rated good, acceptable, poor or failed. (C) Qualitative evaluation of 0.55 T cervix segmentations, rated excellent, good, acceptable or poor. Figure S3: Bar chart showing the external os diameter subtracted from the internal os diameter (mm). Figure S4: Cervical length compared with other cervical measurements: (A) Total cervical volume, (B) Stroma volume, (C) Canal volume, (D) Internal os diameter, (E) External os diameter, (F) Utero‐cervical angle, *p < 0.01. Figure S5: Cervical measurements with gestational age: (A) Cervical length, (B) Internal os, (C) External os, (D) Utero‐cervical angle, (E) Stroma volume, (F) Canal volume. Figure S6: Paired biometry measurements: (A) Cervical length, (B) Stroma volume, (C) Canal volume, (D) Internal os diameter, (E) External os diameter, (F) Utero‐cervical angle. Figure S7: Forest plot with odds ratios for risk of caesarean section with demographics. Figure S8: Forest plot with odds ratios for risk of induction of labour with cervical measurements. Table S1: Definitions for segmentation quality control scoring. Table S2: Formalised measurement definitions for the proposed landmark‐based cervix biometry protocol. Table S3: Demographic data of participants. Table S4: Intraclass correlation coefficients and interpretation for manual cervical measurements by 3 raters. Table S5: Intraclass correlation coefficients and interpretation for average manual and automated cervical measurements. Table S6: MRI automated cervical measurements. Table S7: Change in cervical biometry in paired scans. Table S8: Delivery outcomes. Video S1: Axial, coronal and sagittal reconstructions with overlying segmentations shown in 3D Slicer; purple = outer stomal layer, red = inner stroma layer, blu [file BJO-133-1166-s001.zip › bjo70103-sup-0012-TableS3.docx]

| **Demographics** | **Total (n=92)** |
| --- | --- |
| **Maternal age (years)**  Mean (SD) | 33.4 (4.01) |
| Median [Min, Max] | 34.0 [19.0, 42.0] |
| **Gestational age at MRI (weeks)**  Mean (SD) | 37.2 (1.01) |
| Median [Min, Max] | 37.1 [35.7, 40.1] |
| **Parity** |  |
| Nulliparous | 89 (96.7%) |
| P1 (1 previous caesarean) | 3 (3.3%) |
| **Booking BMI (kg/m2)**  Mean (SD) | 24.0 (3.35) |
| Median [Min, Max] | 23.3 [17.9, 31.5] |
| Missing | 3 (3.3%) |
| **Ethnicity**  Asian | 6 (6.5%) |
| Black | 7 (7.6%) |
| East Asian | 4 (4.3%) |
| Mixed Asian | 3 (3.3%) |
| Mixed Black | 3 (3.3%) |
| Other | 5 (5.4%) |
| White | 64 (69.6%) |
| **Smoking history**  Current smoker | 1 (1.1%) |
| Gave up in last 6 weeks prior to booking | 1 (1.1%) |
| Gave up more than 6 weeks prior to booking | 6 (6.5%) |
| No | 84 (91.3%) |
| **Previous cervical surgery (LLETZ or cone biopsy)** |  |
| No | 87 (94.6%) |
| Yes | 5 (5.4%) |
